# Network pharmacology and molecular docking reveal potential mechanism of esculetin in the treatment of ulcerative colitis

**DOI:** 10.1097/MD.0000000000035852

**Published:** 2023-11-10

**Authors:** Ting Cai, Bin Cai

**Affiliations:** a Department of Nephrology, The Affiliated Wuxi People’s Hospital of Nanjing Medical University, Wuxi People’s Hospital, Wuxi Medical Center, Wuxi, China; b Department of Anorectal Surgery, Wuxi Hospital Affiliated to Nanjing University of Chinese Medicine, Wuxi, China.

**Keywords:** esculetin, molecular docking, network pharmacology, prolactin signaling pathway, ulcerative colitis

## Abstract

Ulcerative colitis (UC) is a chronic inflammatory bowel disease of the colonic mucosa. Esculetin is a type of natural coumarin that has many pharmacological activities such as antioxidant, anticancer, anti-inflammatory, etc. A previous study showed that esculetin improved intestinal inflammation and reduced serum proinflammatory cytokines in UC. The present study aimed to utilize network pharmacology and molecular docking to explore the potential mechanism of esculetin against UC. The potential gene targets of esculetin were predicted through SwissTargetPrediction and Super-PRED web servers. UC-related genes were obtained from DisGeNet, OMIM, and GeneCards databases. The overlap between gene targets of esculetin and UC-related genes were identified as the potential targets of esculetin against UC. The interaction between these overlapping genes was analyzed by the STRING database and the core genes were identified by Cytoscape platform. Gene Ontology and Kyoto Encyclopedia of Genes and Genomes pathway enrichment analysis of the core genes were then performed. And the results of these analyses were further confirmed through molecular docking. A total of 50 overlapping genes were identified as the potential action targets of esculetin against UC. Among them, 10 genes (AKT1, STAT1, CCND1, SRC, PTGS2, EGFR, NFKB1, ESR1, MMP9, SERPINE1) were finally identified as the core genes. The Kyoto Encyclopedia of Genes and Genomes pathway enrichment analysis results showed that the top signaling pathway associated with the core genes of esculetin against UC was the prolactin (PRL) signaling pathway. Molecular docking results showed that esculetin has a strong binding affinity to the core genes, as well as PRL and prolactin receptor. This study suggests that esculetin may have a crucial impact on UC through the PRL signaling pathway and provides insights into the potential mechanism of esculetin in the treatment of UC, which may shed light on the mechanism and treatment of UC.

## 1. Introduction

Ulcerative colitis (UC) is a chronic inflammatory bowel disease of the colonic mucosa characterized by alternating periods of exacerbation and remission.^[1]^ North America and Europe have long been recognized as regions with a high prevalence of UC, with the highest reported prevalence of 505 per 100,000 in Europe (Norway) and 286 per 100,000 in North America (United States).^[2]^ Asia, a region with historically low prevalence of UC, has experienced a striking increase in the 21st century.^[3]^ The exact etiology of UC is not yet fully understood and may involve interactions between the environment, immune system, gut microbiota, and genetic susceptibility.^[4]^

Esculetin (also known as aesculetin and 6,7-dihydroxycoumarin) is a type of natural coumarin extracted mainly from the dried bark of *Fraxinus rhynchophylla* Hance, *Fraxinus chinensis* Roxb., *Fraxinus szaboana* Lingelsh., and *Fraxinus stylosa* Lingelsh.^[5]^ It is one of the simplest coumarin compounds with 2 hydroxyl groups at carbons 6 and 7. Esculetin has been shown to have many pharmacological activities in vitro and in vivo, including antioxidant, anticancer, anti-inflammatory, antidiabetic, anti-atherosclerotic, and so on.^[6–8]^ A recent study demonstrated that administration of esculetin ameliorated intestinal inflammation and reduced serum levels of the pro-inflammatory cytokines such as tumor necrosis factor alpha (TNF-α), interleukin-1β (IL-1β), and IL-6 in a mouse model of dextrose sodium sulfate (DSS)-induced UC.^[9]^ However, the mechanism by which it protects against UC remains to be discovered.

In recent years, network pharmacology has become a widely used tool in drug research to reveal the interrelationships between compounds, targets, pathways, and associated diseases.^[10]^ Molecular docking is a method used to model the interaction between small molecule ligands and proteins at the atomic level, which helps to characterize potential molecular interactions that may occur at the binding site.^[11]^ Based on the research strategy of network pharmacology, our study attempted to predict the core genes and potential signaling pathways of esculetin for the treatment of UC. The affinity between the screened receptors and esculetin was further verified using molecular docking, thus exploring the potential mechanism of esculetin against UC.

## 2. Methods

**Figure 1.**
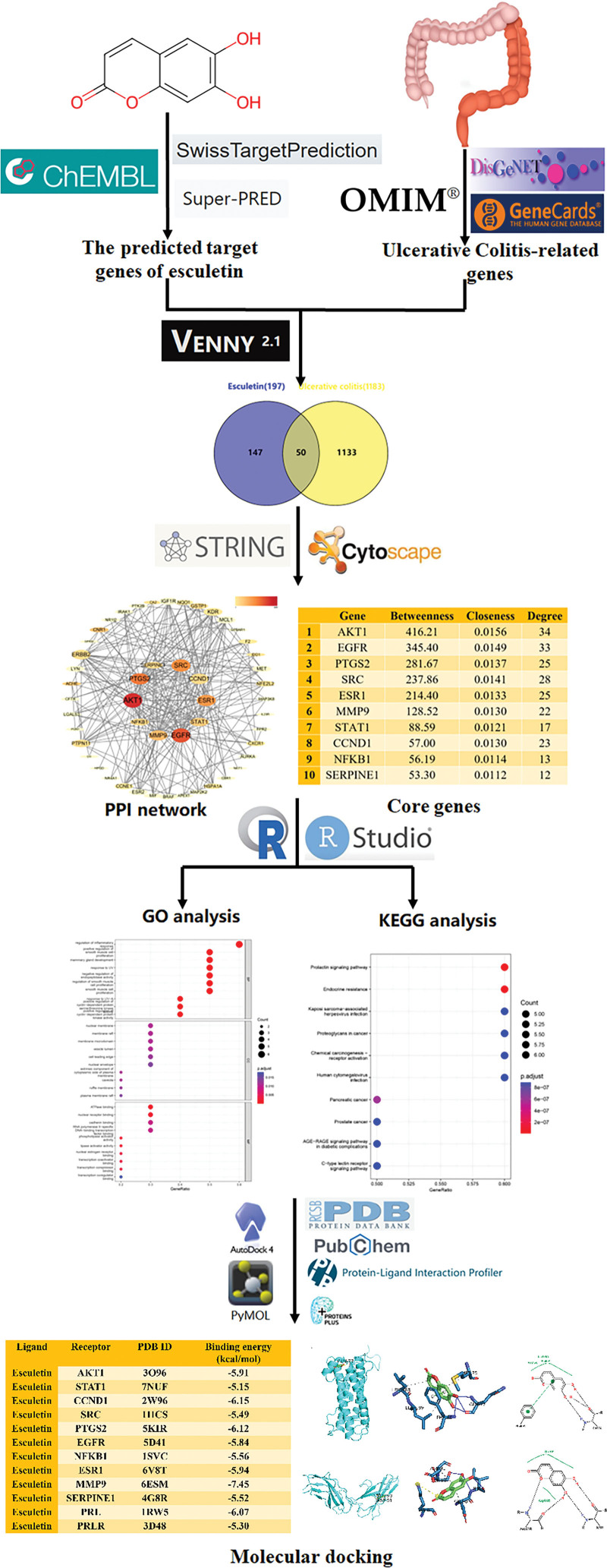
Flowchart of potential mechanism of esculetin in treating ulcerative colitis based on network pharmacology and molecular docking.

The flow chart of the study design was shown in Figure 1.

### 2.1. Screening of the predicted target genes of esculetin

To determine the predicted target genes of esculetin, we first identified the simplified molecular-input line-entry system string of esculetin through the ChEMBL database (https://www.ebi.ac.uk/chembl/), and then we utilized its simplified molecular-input line-entry system string (O = c1ccc2cc(O)c(O)cc2o1) to search the predicted target genes of esculetin in 2 small molecule target prediction databases: SwissTargetPrediction web server (http://www.swisstargetprediction.ch/, accessed on July 21, 2023),^[12]^ and Super-PRED web server (https://prediction.charite.de/, accessed on July 21, 2023).^[13]^ All the target genes were standardized by the UniProt database (https://www.uniprot.org/),^[14]^ and the species was restricted to “*Homo sapiens*” in the search. Final target gene predictions of esculetin were obtained after duplicate removal.

### 2.2. Screening of UC-related genes

The identification of UC-related genes was obtained from 3 well-established databases: DisGeNet (https://www.disgenet.org/, accessed on July 21, 2023),^[15]^ OMIM (https://omim.org/, accessed on July 21, 2023),^[16]^ and GeneCards (http://www.genecards.org/, accessed on July 21, 2023).^[17]^ The search was performed using the term “ulcerative colitis” as the keyword. Genes that appeared in at least 2 of the above 3 databases were identified as final UC-related genes.

### 2.3. Construction of Venn diagram

A Venn diagram was plotted between the predicted target genes of esculetin and the UC-related genes using the online tool Venny 2.1.0 (https://bioinfogp.cnb.csic.es/tools/venny/). These overlapping genes were identified as the potential action targets of esculetin against UC and used for further analysis.

### 2.4. Protein–protein interaction (PPI) network analysis and identification of core genes

A PPI network analysis through the STRING database (https://string-db.org/, accessed on July 21, 2023) was used to analyze the potential action targets of esculetin against UC.^[18]^ The organism was selected as “*Homo sapiens*.” After downloading the data from the STRING database, the PPI network was constructed and visualized using the Cytoscape v3.10.0 platform, and the CentiScaPe 2.2 plug-in was employed for the identification of the core genes.^[19]^ In CentiScaPe 2.2, betweenness, closeness and degree were selected for analysis, and the higher the value, the more important the gene.

### 2.5. Functional enrichment analysis

Gene Ontology enrichment analysis, including Biological Process, Cellular Component, and Molecular Function, and Kyoto Encyclopedia of Genes and Genomes (KEGG) pathway enrichment analysis of the core genes were performed using the clusterProfiler package in R v4.3.0 and RStudio 2023.06. A *P* value of <.05 was considered statistically significant.

### 2.6. Verification of interaction by molecular docking

Molecular docking was performed to predict the binding affinity between esculetin and its potential therapeutic targets for the treatment of UC, which could provide a basis for further experimental verification. The SDF format of esculetin (PubChem CID5281416) was obtained from the PubChem database (https://pubchem.ncbi.nlm.nih.gov/). The PDB formats of the core genes, including AKT1 (PDB ID: 3O96), STAT1 (PDB ID: 7NUF), CCND1 (PDB ID: 5VZU), SRC (PDB ID: 1HCS), PTGS2 (PDB ID: 5F19), EGFR (PDB ID: 5D41), NFKB1 (PDB ID: 1SVC), ESR1 (PDB ID: 6V8T), MMP9 (PDB ID: 6ESM), and SERPINE1 (PDB ID: 4G8R) were downloaded from the PDB database (https://www.rcsb.org/). In addition, the KEGG pathway enrichment analysis concluded that the prolactin (PRL) signaling pathway might play a key role in the treatment of UC with esculetin, which is normally activated by PRL binding to the prolactin receptor (PRLR). Therefore, the PDB formats of PRL (PDB ID: 1RW5) and PRLR (PDB ID: 3D48) were also downloaded. Molecular docking was performed using AutoDockTools v4.2 (http://autodock.scripps.edu/, accessed September 25, 2023).^[20]^ Visualization of AutoDockTools results was performed using PyMOL v2.5 (https://pymol.org/2/), Protein–Ligand Interaction Profiler web server (https://plip-tool.biotec.tu-dresden.de/),^[21]^ and ProteinsPlus webserver (https://proteins.plus/).^[22]^

## 3. Results

### 3.1. Screening of the potential action targets of esculetin against UC

**Figure 2.**
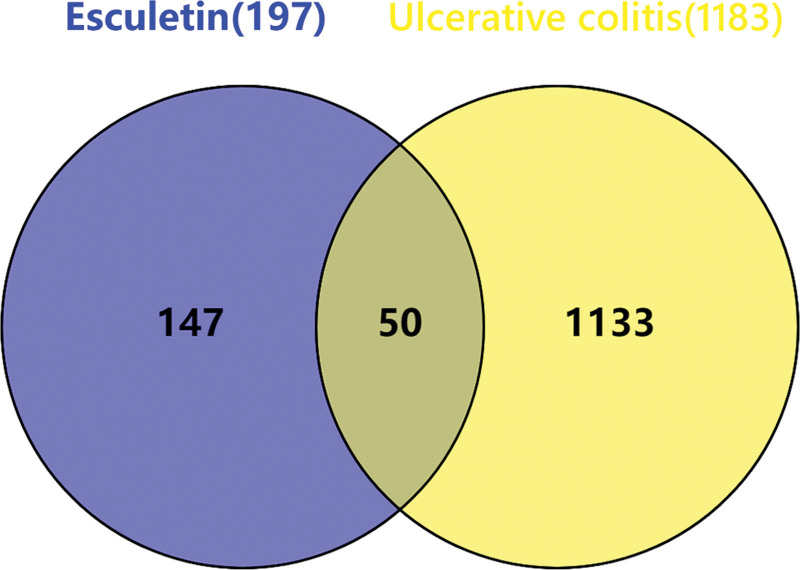
Venn diagram showing the potential action targets of esculetin against ulcerative colitis. A total of 50 overlapping genes were identified as the potential action targets of esculetin against ulcerative colitis.

A total of 100 and 108 predicted target genes of esculetin were obtained by searching the SwissTargetPrediction and Super-PRED web servers, respectively. After removing duplicates, 197 predicted target genes of esculetin were finally obtained. A total of 1458, 192 and 6113 UC-related genes were obtained by searching the DisGeNet, OMIM, and GeneCards databases, respectively. Among them, 1183 genes were present in at least 2 of the 3 databases, and these genes were identified as the final UC-related genes. As shown in the Venn diagram (Fig. 2), a total of 50 overlapping genes were identified as the potential action targets of esculetin against UC.

### 3.2. PPI network and identification of core genes

**Figure 3.**
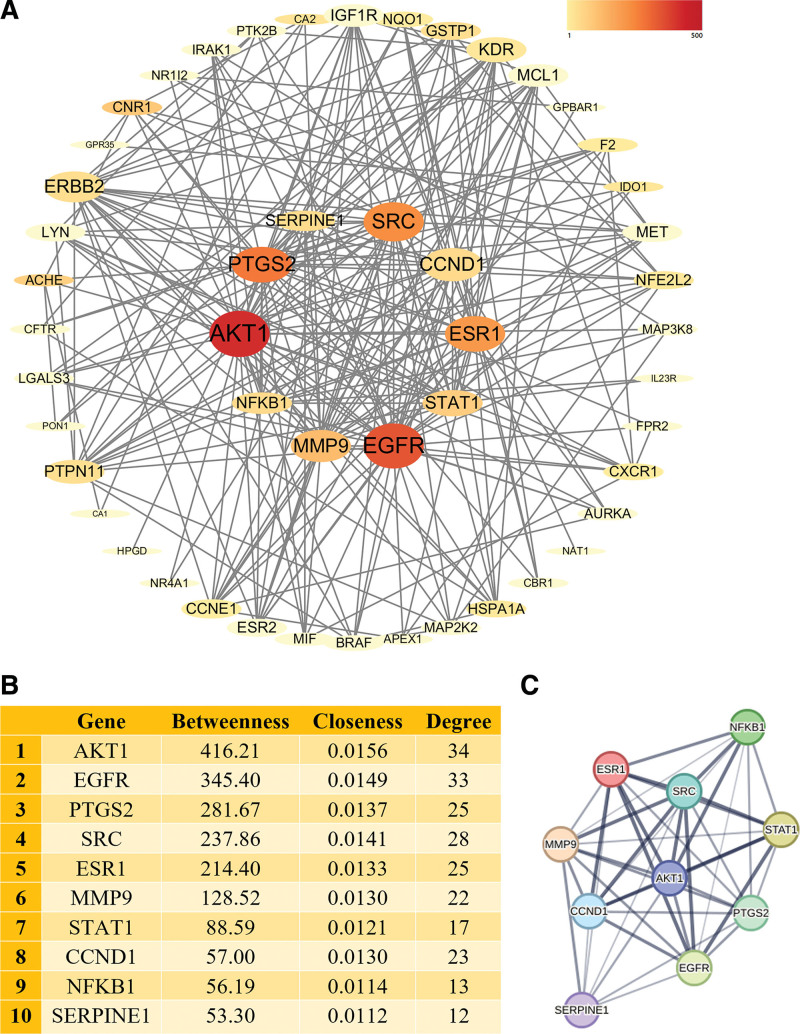
PPI network and core genes of esculetin against UC. (A) PPI network of the potential action targets of esculetin against UC, darker colors, larger fonts, and larger shapes of genes indicate higher betweenness, closeness, and degree values. The 10 genes in the center are the core genes. (B) Betweenness, closeness, and degree values of core genes including AKT1, STAT1, CCND1, SRC, PTGS2, EGFR, NFKB1, ESR1, MMP9, and SERPINE1. (C) PPI network of the 10 core genes, line thickness indicates the strength of data support. UC = ulcerative colitis, PPI = protein–protein interaction.

A PPI network analysis was performed to demonstrate the interaction between the above 50 overlapping genes (Fig. 3A). In CentiScaPe 2.2, genes whose values simultaneously exceeded the betweenness centrality, closeness centrality, and degree centrality were identified as core genes (Fig. 3B). Finally, 10 genes (AKT1, STAT1, CCND1, SRC, PTGS2, EGFR, NFKB1, ESR1, MMP9, and SERPINE1) were identified as core genes. A PPI network of the 10 core genes was constructed using the STRING database (Fig. 3C).

### 3.3. Functional enrichment analysis

**Figure 4.**
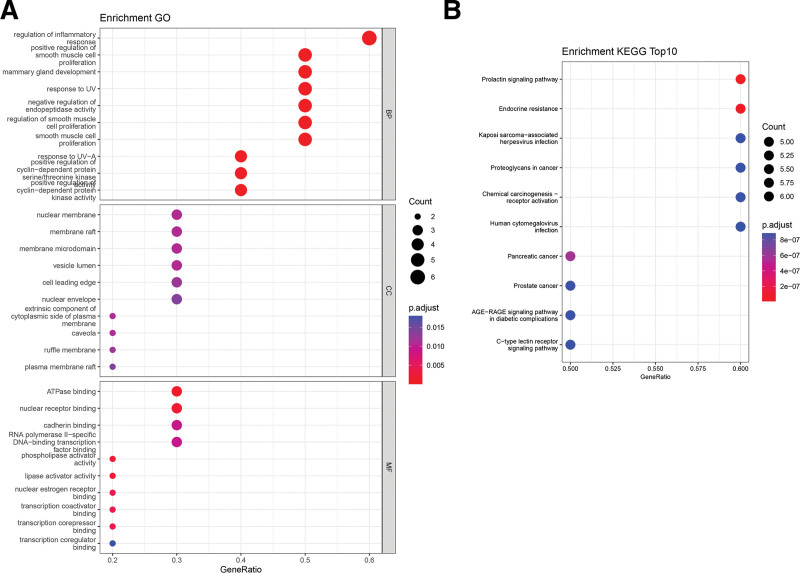
GO and KEGG enrichment analysis. (A) GO enrichment analysis of the core genes of esculetin against UC. (B) KEGG pathway enrichment analysis of the core genes of esculetin against UC (top 10). UC = ulcerative colitis, GO = Gene Ontology, BP = Biological Process, CC = Cellular Component, MF = Molecular Function, KEGG = Kyoto Encyclopedia of Genes and Genomes.

The results of Gene Ontology analysis revealed that the top biological process associated with the core genes of esculetin against UC was related to the regulation of inflammatory response. The Cellular Component analysis revealed that the core genes were primarily involved in the nuclear membrane, membrane raft, and membrane microdomain. In terms of Molecular Function, the top 3 significant enrichment terms were ATPase binding, nuclear receptor binding, and cadherin binding (Fig. 4A). The KEGG pathway enrichment analysis results showed that the top signaling pathway associated with the core genes of esculetin against UC was the PRL signaling pathway (Fig. 4B).

### 3.4. Molecular docking verification

**Figure 5.**
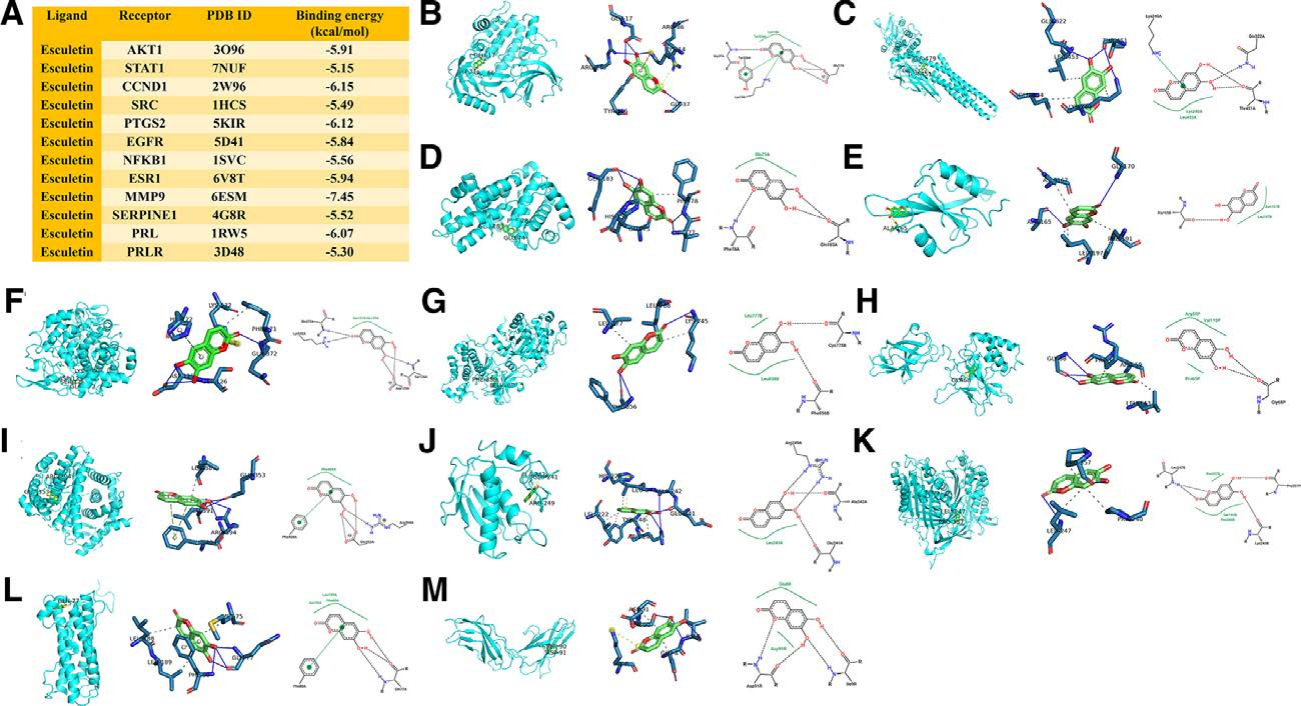
Molecular docking analysis of esculetin and the core genes. (A) The results of ligand-receptor binding energy values. (B) esculetin-AKT1, (C) esculetin-STAT1, (D) esculetin-CCND1, (E) esculetin-SRC, (F) esculetin-PTGS2, (G) esculetin-EGFR, (H) esculetin-NFKB1, (I) esculetin-ESR1, (J) esculetin-MMP9, (K) esculetin-SERPINE1, (L) esculetin-PRL, (M) esculetin-PRLR.

The results of ligand-receptor binding energy values are shown in Figure 5A. The ligand-receptor binding energy represents the stability of the binding, and the lower the binding energy, the stronger the binding force between the ligand and the receptor. It is generally accepted that a binding energy value of less than −5 kcal/mol is considered a stable binding. Molecular docking results showed that esculetin had a strong binding affinity to the 10 core genes (Fig. 5B–K), especially MMP9, CCND1, PTGS2, ESR1, AKT1, and EGFR. Among them, the binding affinity of esculetin to MMP9 was the strongest, with a value of −7.45 kcal/mol. Esculetin formed 4 hydrogen bonds with ARG-249, ALA-242, and GLU-241, and 4 hydrophobic interactions with LEU-222, LEU-243, TYR-248, and ARG-249 in the docking pocket of MMP9 (Fig. 5J). In addition, esculetin also had a strong binding affinity to PRL and PRLR. Esculetin bound to PRL and PRLR with binding energy values of −6.07 kcal/mol and −5.30 kcal/mol, respectively. Esculetin formed 4 hydrogen bonds with GLN-77 and PHE-80, 3 hydrophobic interactions with MET-75, LEU-188 and LEU-189, and a π-stacking with PHE-80 in the docking pocket of PRL (Fig. 5L). Esculetin formed 4 hydrogen bonds with ULE-9 and SER-90, and 2 hydrophobic interactions with GLU-8 and ASP-91 in the docking pocket of PRLR (Fig. 5M).

## 4. Discussion

A recent study has confirmed the potential of esculetin to ameliorate DSS-induced UC by reducing inflammation and regulating cytokine release.^[9]^ Using a network pharmacology approach, our study ultimately identified 10 core genes, including AKT1, STAT1, CCND1, SRC, PTGS2, EGFR, NFKB1, ESR1, MMP9, and SERPINE1, which may be potential targets of esculetin against UC. Furthermore, molecular docking showed that esculetin had a strong binding affinity to the above core genes. Among them, STAT1, PTGS2 (also known as COX-2), and NFKB1 are common inflammation-associated genes that are closely involved in the release of inflammatory mediators and cytokines such as IL-1β, IL-6, interferon gamma, TNF-α, inducible nitric oxide synthase (prostaglandin E2).^[23,24]^ The polarization imbalance of M1/M2 macrophages in UC has been the focus of recent attention. AKT1 is strongly associated with the M1 phenotype of macrophages, which plays a key role in colitis.^[25]^ EGFR is an upstream molecule of the mitogen-activated protein kinase (MAPK) signaling pathway.^[26]^ SRC is one of the major protein tyrosine kinases that regulate the MAPK signaling pathway as well as the nuclear factor-kappa B signaling pathway.^[27]^ Both the MAPK and nuclear factor-kappa B signaling pathways are critically involved in the pathogenesis of UC due to the abundant release of cytokines and inflammatory mediators upon their activation.^[28]^

Matrix metalloproteinases (MMPs) are a family of zinc-dependent proteolytic endopeptidases with extracellular matrix remodeling and degradation properties. Most MMPs are transcriptionally upregulated in response to proinflammatory cytokines, cell-cell, or cell-extracellular matrix interactions.^[29]^ It has been reported that the mucosal expression and serum levels of MMP9 were significantly higher in UC patients compared to the control group, and the increased MMP9 expression contributed to the severity of mucosal damage in active UC.^[30]^ In addition, serum levels of MMP9 were significantly associated with C-reactive protein, white blood count, platelets, and Mayo endoscopic score in UC patients.^[31]^ The role of MMP9 in UC is not well understood and may involve the release of cytokines and chemokines, angiogenesis, regulation of tight junction proteins, goblet cell differentiation, and so on.^[32]^ Our molecular docking results showed that esculetin had a strong binding affinity to MMP9 with a value of −7.45 kcal/mol. It is suggested that esculetin may be a potential therapeutic agent for UC through its interaction with MMP9.

The KEGG pathway enrichment analysis results indicated that the PRL signaling pathway may play an important role in the treatment of UC with esculetin. PRL is a 23 kDa peptide hormone composed of 199 amino acids that is principally secreted by the lactotrophs in the anterior pituitary gland. In addition to the 23 kDa monomeric PRL, 2 other major forms are present in the circulation: 40 to 60 kDa big PRL (dimer of the monomeric form) and >150 kDa big-big PRL (complexes of the monomeric form and IgG autoantibodies). However, the monomeric form of PRL is the most active and the higher molecular weight forms have minimal biological activity.^[33]^ As both a circulating hormone and a cytokine, the primary function of PRL is to promote mammary gland development, lactation, and pregnancy. Aside from its actions on reproductive processes, PRL plays a role in maintaining the constancy of the internal environment by regulation of the immune system, osmoregulation, angiogenesis, and so on.^[34]^ The secretion of PRL is mainly under the inhibitory control of hypothalamic dopamine (DA), and it is also subject to a negative feedback loop of its own. The critical role of DA in suppressing endogenous PRL secretion has been well established. Tuberoinfundibular dopamine neurons located in the dorsomedial arcuate nucleus secrete DA into the pituitary portal blood vessels. The D2 DA receptor is abundantly expressed in the pituitary gland, which mediates the inhibition of PRL secretion.^[35]^ In addition to the pituitary gland, PRL can also be produced in extrapituitary locations, such as decidua, ovary, prostate, mammary gland, adipose tissue, brain, and immune cells.^[36]^

PRL binds to its receptor PRLR, which belongs to the class I cytokine receptor family that has no intrinsic kinase activity.^[37]^ PRLR consists of an extracellular domain, a single transmembrane domain, and an intracellular domain. When PRL binds to PRLR, it causes receptor dimerization, resulting in the initiation of several intracellular signal transduction, including canonical Janus kinase 2/signal transducer and activator of transcription (JAK2/STAT) pathway, MAPK pathway, and phosphatidylinositol-3-kinase/protein kinase B (PI3K/Akt) pathway.^[38]^ JAK2 is a non-receptor tyrosine kinase that is rapidly activated upon stimulation with PRL, promoting the phosphorylation and nuclear localization of STATs, including STAT3 and STAT5.^[39]^ The JAK2/STAT3 pathway is an extremely important intracellular signal transduction pathway that has been widely recognized as a critical modulator of a variety of biological processes, including innate and adaptive immunity and inflammation.^[40]^ Blockade of the JAK2/STAT3 pathway has been demonstrated to modulate innate and acquired immune responses and to attenuate chronic intestinal inflammation.^[41]^ Comparatively, activation of JAK2/STAT5 signaling may exert a protective effect against UC. Phosphorylation of STAT5 promotes the synthesis of Foxp3, which promotes the differentiation of CD4^+^ T cells into regulatory T cells.^[42]^ Regulatory T cells always play a role in immune homeostasis and suppressing inflammatory responses.^[43]^ Furthermore, activated PI3K/Akt pathway is also implicated in the regulation and release of proinflammatory cytokines such as IL-1β, IL-6, IL-8, and TNF-α, which in turn participates in the development of UC.^[44]^

**Figure 6.**
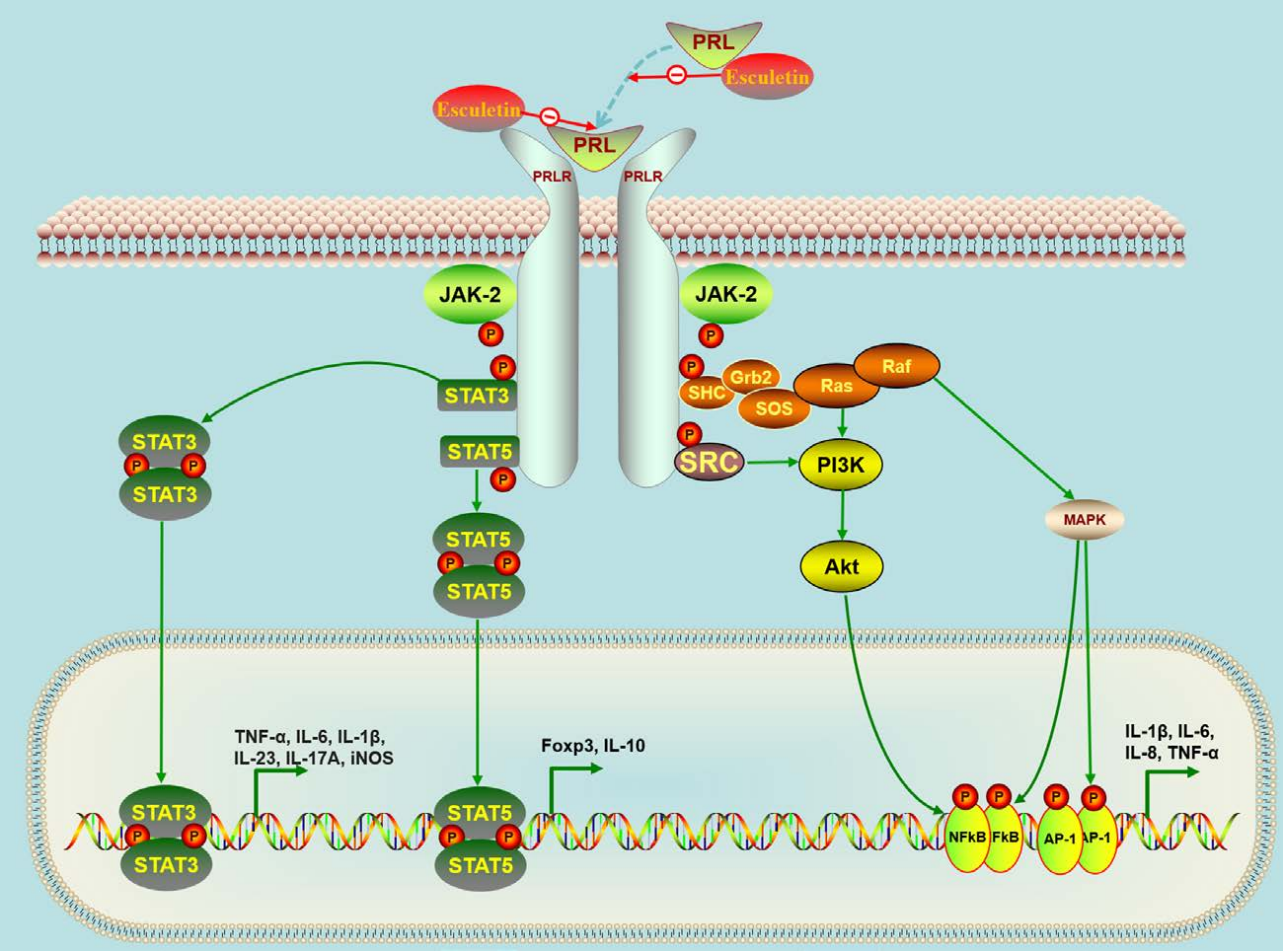
Potential mechanism of action of esculetin against ulcerative colitis via the prolactin signaling pathway. Akt = protein kinase B, AP-1 = activator protein 1, FOXP3 = forkhead box protein P3, IL-1β = interleukin-1β, iNOS = inducible nitric oxide synthase, JAK2 = Janus kinase 2, MAPK = mitogen-activated protein kinase, NF-κB = nuclear factor-kappa B, PI3K = phosphatidylinositol-3-kinase, PRL = prolactin, PRLR = prolactin receptor, Raf = root abundant factor, RAS = rat sarcoma virus, STAT = signal transducer and activator of transcription, TNF-α = tumor necrosis factor alpha.

It was reported that DA levels in the inflamed mucosa of UC patients were markedly lower than in controls.^[45]^ Similarly, decreased levels of DA were detected in the colonic mucosa of TNBS-induced rats,^[46]^ in the colonic mucosa of DSS- induced mice,^[47]^ and in the feces of TRUC (*T-bet^−/−^ Rag2^−/−^* ulcerative colitis) mice.^[48]^ In UC, a decrease in DA levels may lead to a weakening of its inhibitory effect on PRL secretion, ultimately resulting in an increase in PRL levels. A clinical study from China with 72 UC patients and 72 healthy controls showed that UC patients had significantly higher PRL levels than healthy controls (*P* < .01, 28.2 ± 16.7 μg/L vs 12.98 ± 7.7 μg/L).^[49]^ In the present study, by inputting the core genes of esculetin against UC for KEGG pathway enrichment analysis, the main significantly enriched pathway was the PRL signaling pathway. Subsequently, we used molecular docking to predict the binding affinity of the esculetin ligand to PRL and PRLR. The results showed that the binding energy values of esculetin to PRL and PRLR were both <−5 kcal/mol, indicating that esculetin could form stable binding conformations with PRL and PRLR. Since the biological activity of PRL is highest when in a monomeric form, binding of esculetin to PRL may reduce PRL activity. On the other hand, if esculetin competes with PRL for its receptor PRLR, it may block activation of the PRL-induced pathway. This ultimately leads to inhibition of the downstream JAK2/STAT, MAPK, and PI3K/Akt pathways associated with UC. These above results offered an initial theoretical basis for further experimental investigation (Fig. 6).

However, the present study has some limitations. Firstly, the pharmacological mechanism of esculetin in the treatment of UC in our study is based on computational technologies and still requires further validation through pharmacological and clinical studies. Secondly, some reports suggest that MMP9 upregulation is a consequence rather than a cause of intestinal inflammation,^[50]^ so the role of MMP9 in UC remains to be clarified. Thirdly, STAT3 and STAT5, as downstream of the PRL signaling pathway, seem to play opposite roles in UC. This will cause some disagreement on the role of the PRL pathway in UC. Fourthly, the changing profile of PRL expression levels in UC patients need to be supported by more clinical data.

## 5. Conclusion

In summary, our study utilized a network pharmacology approach to investigate the potential mechanism of esculetin in the treatment of UC. Our study ultimately identified 10 core genes, including AKT1, STAT1, CCND1, SRC, PTGS2, EGFR, NFKB1, ESR1, MMP9, and SERPINE1, which may be potential targets of esculetin against UC. The KEGG pathway enrichment analysis indicated that esculetin may have a crucial impact on UC through the PRL signaling pathway. Our molecular docking analysis also showed that esculetin effectively interacted with PRL and PRLR in addition to the 10 core genes. Overall, our study provides a reference for further investigating the mechanism of esculetin against UC. Furthermore, we also want to draw the attention of researchers to the role of PRL signaling pathway on UC, which may shed light on the mechanism and treatment of UC.

## Author contributions

**Conceptualization:** Bin Cai.

**Funding acquisition:** Ting Cai, Bin Cai.

**Methodology:** Ting Cai

**Project administration:** Bin Cai.

**Visualization:** Bin Cai.

**Writing – original draft:** Ting Cai, Bin Cai.

**Writing – review & editing:** Bin Cai
